# Biomolecular Simulations with the Three-Dimensional Reference Interaction Site Model with the Kovalenko-Hirata Closure Molecular Solvation Theory

**DOI:** 10.3390/ijms22105061

**Published:** 2021-05-11

**Authors:** Dipankar Roy, Andriy Kovalenko

**Affiliations:** 1 10-203 Donadeo Innovation Centre for Engineering, Department of Mechanical Engineering, University of Alberta, Edmonton, AB T6G 1H9, Canada; 2 Department of Biological Sciences, University of Alberta, Edmonton, AB T6G 2E9, Canada; 3 Nanotechnology Research Centre, National Research Council of Canada, Edmonton, AB T6G 2M9, Canada

**Keywords:** molecular solvation theory, three-dimensional reference interaction site model, Kovalenko-Hirata closure, biomolecular simulation, multiple time step MD, protein-ligand binding, biomolecular solvation

## Abstract

The statistical mechanics-based 3-dimensional reference interaction site model with the Kovalenko-Hirata closure (3D-RISM-KH) molecular solvation theory has proven to be an essential part of a multiscale modeling framework, covering a vast region of molecular simulation techniques. The successful application ranges from the small molecule solvation energy to the bulk phase behavior of polymers, macromolecules, etc. The 3D-RISM-KH successfully predicts and explains the molecular mechanisms of self-assembly and aggregation of proteins and peptides related to neurodegeneration, protein-ligand binding, and structure-function related solvation properties. Upon coupling the 3D-RISM-KH theory with a novel multiple time-step molecular dynamic (MD) of the solute biomolecule stabilized by the optimized isokinetic Nosé–Hoover chain thermostat driven by effective solvation forces obtained from 3D-RISM-KH and extrapolated forward by generalized solvation force extrapolation (GSFE), gigantic outer time-steps up to picoseconds to accurately calculate equilibrium properties were obtained in this new quasidynamics protocol. The multiscale OIN/GSFE/3D-RISM-KH algorithm was implemented in the Amber package and well documented for fully flexible model of alanine dipeptide, miniprotein 1L2Y, and protein G in aqueous solution, with a solvent sampling rate ~150 times faster than a standard MD simulation in explicit water. Further acceleration in computation can be achieved by modifying the extent of solvation layers considered in the calculation, as well as by modifying existing closure relations. This enhanced simulation technique has proven applications in protein-ligand binding energy calculations, ligand/solvent binding site prediction, molecular solvation energy calculations, etc. Applications of the RISM-KH theory in molecular simulation are discussed in this work.

## 1. Introduction

The developments of molecular simulations started first with statistical methods like Monte-Carlo simulations (MC) to address the time-progression of multi-particle systems. The use of macroscopic spheres to simulate atomic motions dates backs to early 1940. The work on elastic collision in phase transition by Alder and Wainwright is attributed as the first realistic simulation [1]. The progress in molecular dynamics (MD) simulations from that point is phenomenal thanks to ever evolving computer architectures and development of efficient algorithms. While the initial applications of the MD simulations were aimed at material science applications, they covered biophysics and biomolecules very fast. The applications in biomolecular systems are numerous: X-ray structure processing, protein folding, and receptor-ligand interactions, to name a few [2,3,4]. Performance and accuracy of MD simulations were verified by comparison with experimental data obtained from diffraction and NMR experiments. An essential component of MD simulation, the force field is developed by fitting against high-level quantum chemical calculations [5,6,7]. The other variant of the force field, the Kirkwood-Buff (KB) force fields designed through application of the KB theory in calculating densities and other physical properties of multicomponent solutions, has shown immense potential for use in molecular simulations, both with all atom and united atom settings [8,9,10]. The deviations of MD simulations from experimental results are attributed to the shortcomings of the force field(s) used, as well as inadequate simulation time frame. It is imperative to point to the local minima problem faced by MC and MD methods for potential energy surfaces (PESs) with multiple minima separated by large energy barriers [11,12,13]. A plethora of research has been devoted to overcome such issues [14,15]. The explicit solvent simulations using MD techniques are the most adequate ones for modeling biologically important molecules which often requires specific environments. Explicit solvation simulations are quintessential for solvation free energy as well as receptor-ligand binding energy calculations. All these theoretical and computational techniques essentially deal with multiple interactions present in liquid environment (e.g., solution). These interactions involve solvent-solvent and solute-solvent interactions. The solute-solvent interaction further breaks down into the electrostatic and non-polar components. To complete the interaction terms in order to calculate solvation energy, solute polarization and deformation energies are important factors. The last term becomes more significant for binding studies in biomolecular simulations. Incorporating all these intra- and intermolecular terms in solvation process modeling is a daunting task, and justifies development of several theoretical methods to address molecular solvation.

The differences in molecular properties between isolated systems and continuum calculations are often the results of different scalabilities of the systems under consideration. The “gold-standard” quantum mechanical (QM) methods can achieve accuracy up to one-tenth of a kcal/mol, but limited to systems with small sizes [16,17,18,19]. Different continuum solvation models (e.g., PCM and its variants, SMD, COSMO) are calibrated against experimental solvation energy databases of small molecules, and are problematic for absolute solvation energy calculations of systems beyond the chemical classes covered in calibration databases (viz. transition metal containing systems) [20,21,22,23,24]. The difficulty in achieving accurate solvation free energy prediction can be attributed to the absence of specific solute-solvent interactions, limited (or most of the time, absent) sampling of the solute conformal steps, etc. Application of quantum chemical calculations of biomolecules (protein, DNA/RNA) are restricted due to system size resulting in a large number of basis functions required to describe such systems. The ONIOM methodology as well as QM/MM and QM/MM/MD techniques provide respite to this handicap by offering a computationally more amenable scenario where site(s) of importance are treated with high level QM calculations while the rest of the system is treated with molecular mechanics potentials for specialized applications [25,26,27,28].

**Figure 1:**
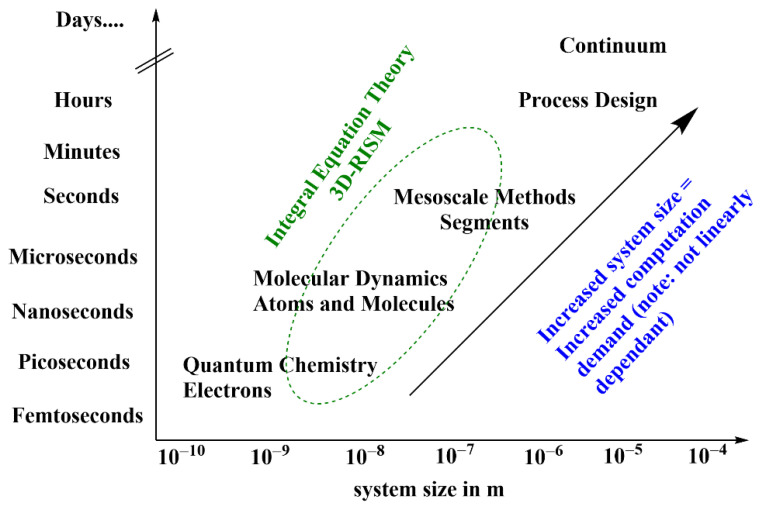
Computational simulation scale and versality of the 3D-RISM theory.

The applicability of different theoretical methods to solvation dynamics of systems of different sizes and dimensions is quite compartmentalized. Thus, a theoretical model that spans over a large scale of computational requirements with reasonable accuracy and speed is desirable, and much research activity is devoted toward this goal. The reference interaction site model (RISM) is based on first principle statistical mechanics, with proven applications in the field of van der Waals fluid, biomolecules, material science, and drug development [29,30,31]. The theoretical framework of RISM is suitable to couple with MD-engines and QM self-consistent-field (SCF) iterations [32,33,34,35]. This makes the RISM formalism an excellent candidate from the perspective of building materials of desired properties, as the theory provides understanding of all the underlying interactions between different constituent fragments. The RISM theory with the integral equation formalism was developed and used for solvation structure and energetics calculations, although the potential of this theory goes beyond regular solvation energy calculations and expands to molecular partitioning, physical-chemical property calculation, and molecular simulations [36,37,38,39,40,41,42,43,44,45,46,47,48]. The key feature of the RISM theory is that it can provide reasonably accurate result rapidly, a feature that made this the theory an essential part of the multiscale modeling framework (Figure 1).

## 2. Theoretical Background

**Figure 2:**
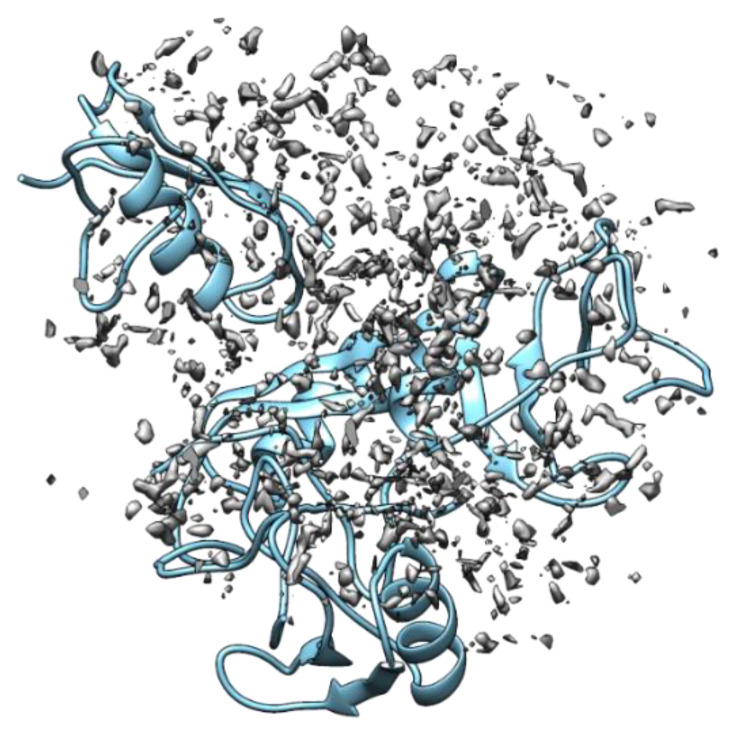
The normalized distribution of the water oxygen sites around the scorpion toxin protein (PDB ID: 1AHO) computed using the 3D-RISM-KH theory and the modified TIP3P water model. The protein backbone is colored in cyan.

The foundation of the RISM theory is credited to the seminal works of Chandler and coworkers [49,50,51,52,53,54,55,56]. This theory grew enormously over past forty years. The key theoretical aspects are outlined in this section. Further theoretical backgrounds are provided for individual applications in the respective sections. For a solute of arbitrary shape, the 3-dimensional (3D-) version of the RISM theory provides a probability distribution of all possible interaction sites (*γ*) of solvent molecules around the solute at position **r** which is a product of the average number density (*ρ*_*γ*_) in the bulk solution and the normalized density distribution, *g*_*γ*_(*r*) (Figure 2). The density enhancement and/or depletion (*g*_*γ*_(*r*) > 1 and/or *g*_*γ*_(*r*) < 1) relative to the average density at a point in solution bulk where g_*γ*_(*r*) → 1 is provided by the average number density. The total correlation function of solvent sites in 3D is related to the 3D direct correlation function *c*_*γ*_(*r*) and site-site bulk susceptibility function for α-solvent sites around a solute by Equation (1).

(1) $$h_{\gamma }\left(r\right)=\underset{\alpha }{\sum }\int dr^{\prime }c_{\alpha }\left(r-r^{\prime }\right)\chi_{\alpha \gamma }\left(r^{\prime }\right)$$

Additionally, *g*_*γ*_(*r*) = *h*_*γ*_(*r*) + 1 and *c*_*γ*_(*r*) ~ −*u*_*γ*_(*r*)/(*k_B_T*), where *T* is temperature and k_B_ is the Boltzmann constant. The bulk susceptibility function *χ* is an essential input to the 3D-RISM integral equation, and is constructed from the intramolecular correlation function *ω*_α__*γ*_ from the dielectrically consistent RISM (DRISM) [57]:

*χ*_α__*γ*_(*r*) = *ω*_α__*γ*_(*r*) + *ω*_α__*γ*_(*r*) *ρ*_*γ*_ *h*_α__*γ*_(*r*) (2)

The intramolecular correlation function can be expressed in reciprocal k-space via terms of a zeroth-order Bessel function:

(3) $$\omega_{\alpha \gamma }\left(r\right)=j_{0}\left(kl_{\alpha \gamma }\right)$$

The intra- and inter-species correlation functions are renormalized through an analytical dielectric bridge function for solvents with high dielectric constant value, thus ensuring that all inter- and intra-species interactions are considered for a few solvent layers around a solute (or cosolvent, etc.). The renormalized form of the dielectric correction is written in terms of zeroth- and first-order Bessel functions over the position of each atom *r*_α_ = (*x_α_*, *y_α_*, *z_α_*) with partial charge *q_α_* of site *α* on species with respect to its molecular origin:

(4) $$\chi_{\alpha \gamma }\left(k\right)=j_{0}\left(kx_{\alpha }\right)j_{0}\left(ky_{\alpha }\right)j_{1}\left(kz_{\alpha }\right)h_{c}\left(k\right)j_{0}\left(kx_{\gamma }\right)j_{0}\left(ky_{\gamma }\right)j_{1}\left(kz_{\gamma }\right)$$

The envelope function *h*_c_(*k*) determines the dielectric constant of the solution using a non-oscillatory form with amplitude A falling off rapidly at wavevectors *k* larger than the characteristic size *l* of the liquid. The characteristic length is important for DRISM calculations, to avoid spurious non-physical distribution functions.

(5) $$h_{c}\left(k\right)=A\exp\left(-l^{2}k^{2}/4\right)$$

(6) $$A=\frac{1}{\rho_{\mathrm{polar}}}\left(\frac{\varepsilon }{y}-3\right)$$

For a mixed solvent scenario, the total number density of polar species and solution dielectric susceptibly *y* can be used in combination with Equations (4)–(6) to apply 3D-RISM formalism as:

(7) $$\rho_{\mathrm{polar}}=\underset{s\in \mathrm{polar}}{\sum }\rho_{s}$$

(8) $$y=\frac{4\pi }{9k_{B}T}\underset{s\in \mathrm{polar}}{\sum }\rho_{s}{\left(d_{s}\right)}^{2}$$

A closure function is required to integrate an infinite chain of correlation diagrams generated from the direct and total correlation function. The functional form of such a closure function in unknown, and several approximated forms were reported over time for simplified computations. Closure functions differ from each other in the mathematical form of the bridging function used in the construct. The Kovalenko-Hirata (KH) closure is among the best closure relations till date in terms of both numerical stability and reasonable accuracy [58,59]. The mathematical form of the KH closure is given as:

(9) $$g_{\gamma }\left(r\right)=\{\begin{array}{ccc} \exp\left(-u_{\gamma }\left(r\right)/\left(k_{B}T\right)+h_{\gamma }\left(r\right)-c_{\gamma }\left(r\right)\right) & \mathrm{for} & g_{\gamma }\left(r\right)\leq 1\\ 1-u_{\gamma }\left(r\right)/\left(k_{B}T\right)+h_{\gamma }\left(r\right)-c_{\gamma }\left(r\right) & \mathrm{for} & g_{\gamma }\left(r\right)>1 \end{array}$$

The overall form of the KH closure can be explained as a coupling of the mean spherical approximation for the regions of density enrichment (*g*_*γ*_(*r*) > 1) with the hypernetted chain approximation for the region of density depletion (*g*_*γ*_(*r*) < 1). The excess chemical potential and the solvation free energy is obtained from the analytical form of the KH closure as:

(10) $$\begin{array}{c} \mu_{\mathrm{solv}}=\underset{\gamma }{\sum }\;\int_{V}dr\;\Phi_{\gamma }\left(r\right)\\ \Phi_{\gamma }\left(r\right)=\rho_{\gamma }k_{B}T\left[\frac{1}{2}h_{\gamma }^{2}\left(r\right)\Theta \left(-h_{\gamma }\left(r\right)\right)-c_{\gamma }\left(r\right)-\frac{1}{2}h_{\gamma }\left(r\right)c_{\gamma }\left(r\right)\right] \end{array}$$

The *Φ*_*γ*_(*r*) is the Heaviside step function. Important thermodynamic parameters are derived from the excess chemical potential for solute sites (u) and solvent sites (v) as:

Δμ = Δε^uv^ + Δε^vv^ − TΔs_V_ (11)

The entropy (ΔS_V_) and partial molar volume (PMV, Ṽ) are calculated as:

(12) $$\Delta S_{V}=-\frac{1}{T}{\left(\frac{\partial \Delta \mu }{\partial T}\right)}_{V}$$

(13) $$\tilde{V}=k_{B}T\chi_{T}\left(1-\underset{\gamma }{\sum }\rho_{\gamma }\int dr\;c_{\gamma }\left(r\right)\right)$$

The errors in 3D-RISM calculations have several origins. Firstly, the internal pressure calculated in the 3D-RISM molecular solvation theory is wrong. A few correction schemes were developed to counter this error [60,61]. Another source of errors arises from the choice of the Lennard-Jones potential used for calculating interaction potentials. A careful calibration is warranted while selecting a force field for a specific application. For example, the computational framework of 3D-RISM failed to converge for polar protic hydrogen atom (e.g., water) with conventional force fields, as the hydrogen atoms has no van der Walls parameters assigned. This problem is circumvented by using a non-zero van der Wall’s terms for hydrogens [62,63]. The KH closure is known to shift the strongly associated peaks while broadening them simultaneously; interestingly, this provides an adequately correct solvation structure.

## 3. Biomolecular Simulations with the 3D-RISM-KH Molecular Solvation Theory

The center of simulations for biophysics related problems are structure-function features of protein and nucleic acids. The structural landscape of biomolecular folding is a high demand research field. Recent achievements in achieving millisecond time scale simulation of protein structure opened further developments in order to explore the entire folding landscape of proteins of reasonable sizes [64,65,66]. The molecular dynamics simulation with the 3D-RISM-KH theory was first incorporated in the AMBER MD simulation suite, using the Sander engine as well as standalone unit for single point solvation free energy calculations [32]. The standard Sander implementation was modified to support long time scale simulations using damped Langevin dynamics for a canonical ensemble to address the instability of the multiple time step MD (MTS-MD). This is achieved by combining two simulation cycles for two different parts of the system (MTS-MD). The outer time steps are obtained from 3D-RISM-KH calculation. For each inner step, the effective solvation force is used to extrapolate solvation force coordinates, based on the outer time steps. The force matrix {F}^(*k*)^ working on each solute atom is approximated as a linear combination of forces at *N* previous steps, at any given time step *t_k_*:

(14) $${\left\{F\right\}}^{\left(k\right)\;}=\overset{N}{\underset{i=1}{\sum }}a_{ki}{\left\{F\right\}}^{\left(i\right)},\;i\in 3D-\mathrm{RISM}\;\mathrm{steps}$$

The weighted coefficients *a_ki_* for a given time step are obtained from the best projection of *N* previous steps. These non-conservative potentials provided a smooth transition between steps. However, strong coupling through the Nosé-Hoover chain of thermostats impeded structural transitions. The next generation of development provided the advanced solvation force extrapolation scheme (ASFE). This development used the optimized isokinetic Nosé-Hoover thermostat (OIN) for each atom by imposing kinetic energy constraints. The fast-dynamics (solute-solute) and slow dynamics (solute-solvent via 3D-RISM) are separated in the ASFE implementation. The accuracy of extrapolation was estimated by relative mean square deviation of the extrapolated effective solvation forces from their original values calculated from converged 3D-RISM-KH for the outer time step. The applicability of the novel formalism containing two separate time cycles for MD simulation of solute-solvent systems were validated against conformational space of alanine dipeptide in water. The subsequent developments, generalized solvation force extrapolation (GSFE), used rotational transformation of the relative coordinates for each atom in order to smoothen the force matrix described previously. In this new development which also used OIN thermostats, a weighting function was introduced for each discretized space. The new algorithm also takes into account that the nearest neighbors have maximum effect on mean solvation forces for any given atom. The efficiency of this new algorithm was shown by the MTS-MD/OIN/GFSE/3D-RISM-KH simulation of a miniprotein (PDB: 1L2Y) and protein G with their reported folded forms [67,68,69]. The miniprotein folding was achieved via the MTS-MD formalism, starting from a fully extend denatured state, at about 60 ns simulation in comparison to the average physical folding time in the order of μs observed via experiment [68].

## 4. Binding Site Mapping

Receptor-ligand binding is in the heart of early-stage drug discovery. A correct mapping helps to stop waste of resources, both financially and computationally. Traditionally, lead-like molecules are used to find potential binding site(s) on a receptor surface. An alternative option to this is fragment-based mapping. These processes will lead to a set of fragments/probe molecules that are potential binders on a receptor surface with defined binding sites [70,71]. Chemical linking based on available linker databases and knowledge of chemical space yields potential leads. The success of this process depends on correctly finding a binding site, usually using empirical scoring algorithms. The 3D-RISM-KH theory essentially provides a 3D-distribution of solvent sites around a solute of arbitrary shape. Thus, one can replace the solvent with a small molecule fragment and even a mixture of fragments, and develop a distribution of unique sites from a mixture of fragments, around a solute of interest. The concept behind this process is easy to visualize, but requires specialized algorithms that can reduce computational burden and help in finding a physically meaningful solution. The 3D-distribution function for ligand site *γ* is given as:

(15) $$g_{\gamma }\left(r_{\gamma }\left(R,\Omega \right)\right)=\int g_{\gamma }\left(r\right)\rho_{\gamma }\left(r-r_{\gamma }\left(R,\Omega \right)\right)dr$$

The ligand sites spatial position is defined with three cartesian coordinates (*R*, translational) and three Euler angles (𝛺, rotational). In practice, the ligand site density distribution is described using a Gaussian-type function. The so-called “site-integrated” potential of mean force (W, SI-PMF) is used to find the most probable binding site(s) of a ligand probe on a receptor [72]:

(16) $$W\left(\Delta \vec{R,}\;\Omega \right)=-k_{B}T\underset{\gamma }{\sum }ln\;g_{\gamma }\left(r_{\gamma }\left(\vec{R},\;\Omega \right)\right)$$

The latest development of this concept used spatial distribution function of a ligand around a protein and thus explored all possible binding modes of the ligand, and final filtering was done based on a scoring function [73]. This scoring function is based on estimated free energy terms and is written as:

(17) $$W_{SP}\left(\left\{r\right\};\Omega \right)\;\approx -RT\;ln\left[\underset{i}{\prod }g_{i}\left(r_{i};\Omega \right)\right]$$

These methodologies were validated against several small molecule binders and protein-ligand datasets [72,73,74].

**Figure 3:**
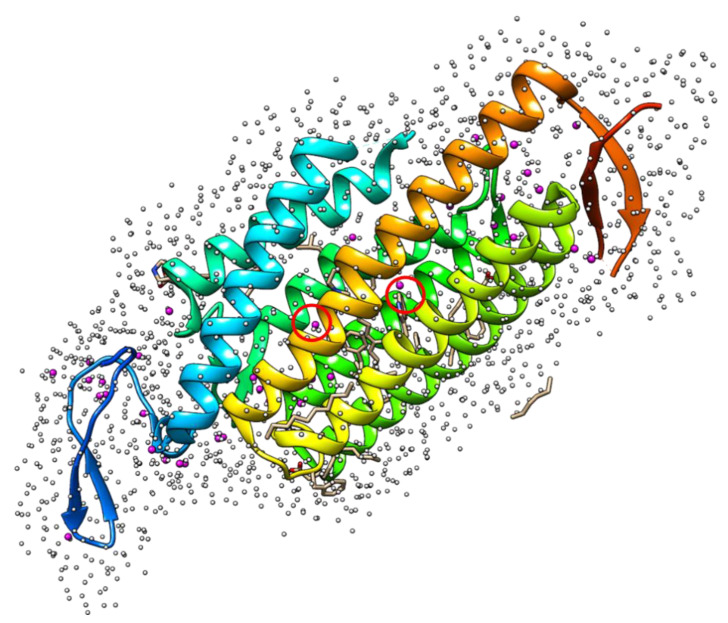
Distribution of water oxygen atoms from 3D-RISM-KH calculations on protein 3UG9 (white spheres). The crystallographic waters are represented with magenta spheres. The catalytic binding site waters were marked in red circle.

Another important aspect of protein-ligand interactions is the role played by binding-site water molecules in ligand recognition [75,76,77]. For a regular molecular simulation with explicit solvent molecules, it is cumbersome to look for such binding site water molecules. This search of binding site waters can be eased with the help of the 3D-RISM-KH water distribution function around a solute molecule. Water distribution in the Lysozyme cavity was first successfully explored to locate binding site water using the 3D-RISM-KH theory [78]. The most updated protocol was reported by Sindhikara and Hirata [79,80]. In their method (placevent), a new successive orthogonal image (SOI) technique for sampling was employed to analyze the distribution function (Figure 3). The SOI method calculates the rotational space of three orthogonal vectors for a spherical search space by using a heavy atom of the solvent as anchor of rotation (e.g., oxygen atoms of water molecules). The solvation site volume (Ṽ*n*) is calculated by applying the Kirkwood-Buff equation in a 3D-RISM-KH calculation as:

(18) $${\tilde{V}}_{n}=k_{B}T\chi_{T}\left(1-\rho_{0}\underset{\gamma }{\sum }\overset{}{\underset{V_{n}}{\int }}c_{\gamma }\left(r\right)\;dr\right)$$

The success of this applications was reported against experimentally determined binding site and poses of ligands in biologically relevant targets. The 3D-RISM-KH based water site prediction is implemented in the MOE^©^ suite [81]. Some examples of successful applications of the 3D-RISM-KH theory in exploiting the explicit role of water maps are reported for in-drug design and protein aggregation studies [82,83,84]. A very recent modification of the 3D-RISM theory in mapping solvation sites in enzyme active site was reported by Nguyen et al. [85]. This new development extended the GIST (grid inhomogeneous solvation theory) based mapping technique in to the 3D-RISM grids. Briefly, an approximated distribution of oxygen site 𝛼 (from water) around a site of interest is related to thermochemical property of interest (*A*) as position r as:

(19) $$A\left(r\right)\;\approx \;A_{\alpha }\left(r\right)+g_{\alpha }\left(r\right)\underset{\gamma \neq \alpha }{\sum }\omega_{\alpha \gamma }\left(r\right)*A_{\gamma }\left(r\right)$$

The number density distribution *g*_α_(*r*) is used to weight the convolution (*) in the right-hand side of Equation (19). This formalism did not consider the non-local effect in the distribution, although the authors reported negligible errors in the final distribution resulting from this issue in comparison to molecular simulations and experimental data.

Among other reports of biomolecular simulations using the 3D-RISM-KH theory, the effect of (micro-) solvent environments on amyloid structure and potential of mean force calculations of solute permeation across UT-B and AQP1 proteins provided further extension of the applications of the 3D-RISM-KH theory based molecular solvation [86,87].

## 5. Protein-Ligand Binding Energy

Heart to drug development is correct prediction or binding affinity of small molecules toward target receptor, if not quantitatively accurate then a trend of binding affinity. Methodologies developed for calculating binding energy for the process, Protein + Ligand → Complex, are the linear-response approximation (LRA), protein-dipole Langevin-dipole approach (PDLD), linear interaction energy (LIE) approach, and MM/PB(GB)SA approach [88,89,90,91,92,93]. The MM/PBSA method is favored over the others, as it avoids empirical parameterizations. In this method, the free energy of binding (*G*_bind_) is expressed via the molecular mechanics (MM) based energy (*E*_MM_, gas phase, for reactants covering internal, electrostatic, and van der Wall’s terms), the solvation energy term (*G*_solv_), and the entropy term (-*T*S_MM_), computed at temperature T.

*G*_bind_ = *E*_MM_ + *G*_solv_ − *TS*_MM_ = *E*_int_ + *E*_el_ + *E*_vdw_ + *G*_solv, polar_ + *g*_solv, non-polar_ − *TS*_MM_ (20)

The solvation terms are calculated by solving the Poisson-Boltzmann (PB) equation (or via generalized Born, GB, model). While this method is fast enough to estimate binding energy in complex, the detailed inter- and intra-species interactions are not transferred properly due to the use of implicit solvation method(s). In the 3D-RISM-KH based binding energy calculation method, the MM/PBSA part is replaced with the 3D-RISM-KH calculations using solvent distribution functions around solutes in the MD simulation trajectory. The PB/GB polar and solvent accessible surface area (SASA) nonpolar solvation terms are replaced with the solvation free energy term from Equation (10). This modification was shown to be equally effective as of traditional MM-PBSA or MM-GBSA methods. The most notable difference was reported between the 3D-RISM-KH and SASA computed non-bonded terms. The later was found to be always favoring binding, whereas the former was not [91,94]. The 3D-RISM-KH based binding calculations were also reported for other protein-ligand complexes and host-guest complexes [95,96,97].

## 6. Molecular Solvation Energy Calculations

The excess chemical potential obtained from 3D-RISM-KH calculations are theoretically a direct measure of solvation energy. However, as mentioned previously, due to erroneous calculations of internal pressure, the computed solvation energy (Gaussian fluctuation excess chemical potential) showed large deviation from actual experimental solvation energy. Other possible reasons for deviations in calculated solvation energy in the 3D-RISM are approximate nature of the closure relations, absence of explicit cavitation energy terms, and inadequacy in force field terms, etc. Incomplete sampling of solutes and short simulation times are also responsible in errors in solvation energy calculations, which reflects in physical property calculations. A correction scheme was developed in order to address this shortcoming, initially for solvation free energy [98]. This so-termed universal correction has the form:

Δ*G*_hydration_*=* Δ*G*^GF^_hydration_, _RISM_ + *a* × PMV + *b* (21)

The partial molar volume (PMV) of a solute in water is an output of a RISM calculation. The coefficients *a* and *b* were obtained from regression analysis against the experimental solvation energy database of Mobley and co-workers [99]. For hydration energy calculations with the 3D-RISM-KH formalism, a modified correction scheme was reported by Truchon et al. which aimed to account for the cavitation energy term [100]. Further development of solvation energy calculations in various solvents were reported from the lab of the authors, both in the context of exploring liquid state of pure solvents as well as in calculating molecular partitioning and permeability properties. Effect of atomic charge assignment schemes in hydration free energy calculation was reported by Roy et al. for an extended database of compounds with experimental hydration free energy [37]. Literature reports on hydration free energy calculations with the 3D-RISM-KH theory obtained excellent results with the GAFF force field parameters of the solute with the modified point charge models of water. While water is one of the most polar solvents used in biochemical simulations, the RISM-KH theory is extended to non-polar solvents too. Non-polar solvents that are modeled using the 3D-RISM-KH theory are hydrocarbons (hexadecane, cyclohexane), haloalkane (chloroform), and alcohol (n-octanol, t-butanol) [41,47,101,102]. Other solvents like nitro-compounds (nitromethane, nitroethane, and nitrobenzene), acetonitrile, and dimethyl sulfoxide (DMSO) were also used in RISM-KH calculations for both liquid structure and solvation energetic studies [42,103,104]. The performance of the 3D-RISM-KH calculations is summarized in Table 1. The performance of the 3D-RISM-KH theory in solvation free energy calculation, in comparison to the performance of MD and/or quantum chemical models, together with computation speed made this theory ideal for such applications.

## 7. Conclusions

The 3D-RISM theory is under continuous development, and the range of the application of this theory is ever expanding. For biomolecular simulations, MTS-MD provides a platform to combine fast dynamics of the solute with slow solute-solvent dynamics, and is proven to be able to avoid the local minima problem in molecular dynamics. Several important modifications covering the algorithm of the 3D-RISM code for application with massive parallel computer architectures were reported [106,107,108]. The algorithm was also coupled with density functional theory based electronic grids for electronic structure calculations [33,109]. It is important to understand that 3D-RISM-KH molecular solvation theory deals with liquid state. Thus, a direct comparison of the simulation results obtained from 3D-RISM calculations with structures determined from solid state experiments (e.g., solid-state X-Ray, neutron diffraction, etc.) may not result in a great match. For a better comparison, data obtained from experiments with liquid state should be used. Further, the Gaussian fluctuation excess chemical potentials from a 3D-RISM calculation should not be taken as an absolute measure of solvation free energy. For solvation energy calculations, the results should be compared against experimental datasets and should be fitted for use against a test set, should such a need arise. The 3D-RISM calculations provide a unique machinery to represent liquid medium with specific concentrations of cosolvent(s), additives, etc., and thus providing an opportunity to model a more realistic environment. The theory is extendable to multiphasic systems with inhomogeneous version of molecular solvation theory. However, one should keep in mind that the 3D-RIM-KH theory is not one a “size fits all” theory. It requires detailed benchmarking of every aspects of a specific problem before using it for predictive modeling.

## Figures and Tables

**Table 1:** Performance of the 3D-RISM-KH theory in predicting solvation free energy of solutes in various solvents reported in the literature. Performance of different computational method in solvation free energy calculation is provided in parentheses.

Solvent	Dielectric Constant	No. of Solutes	Accuracy (Kcal/Mol)	Reference
Water	78.5	504	0.91–0.95 ^*a*^ (1.51) ^*c*^	[99]
			0.89 ^*a*^	[100]
n-Octanol	9.86	205	0.94 ^*b*^	[41]
		158	1.03 ^*b*^	[102]
Cyclohexane	2.0165	91	1.12 ^*a*^	[37,101]
Hexadecane	2.0402	189	0.88 ^*a*^	[37,101]
Chloroform	4.7113	105	0.75 ^*a*^	[37,101]
Acetonitrile	35.688	7	2.2 ^*a*^ (1.9) ^*d*^	[47]
Nitromethane	36.562	7	1.32 ^*a*^ (1.83) ^*d*^	[103]
Nitroethane	28.29	7	0.38 ^*a*^ (2.00) ^*d*^	[103]
Nitrobenzene	34.809	15	0.88 ^*a*^ (2.91) ^*d*^	[103]
DMSO	46.826	8	2.09 ^*a*^	[42]

*^a^* Mean absolute error. *^b^* Relative mean square error. *^c^* RMSE computed from MD simulation in ref. [99]. ^*d*^ RMSE computed using CPCM continuum solvation model on Minnesota solvation database [105].

## Data Availability

All data are available in the original research articles referenced in this review.
